# *Listeria monocytogenes*, a down-to-earth pathogen

**DOI:** 10.3389/fcimb.2013.00087

**Published:** 2013-11-28

**Authors:** Anne-Laure Vivant, Dominique Garmyn, Pascal Piveteau

**Affiliations:** ^1^UMR1347 Agroécologie, Université de BourgogneDijon, France; ^2^UMR1347 Agroécologie, INRADijon, France

**Keywords:** *Listeria*, soil, contamination, occurrence, biodiversity, persistence, circulation, environment

## Abstract

*Listeria monocytogenes* is the causative agent of the food-borne life threatening disease listeriosis. This pathogenic bacterium received much attention in the endeavor of deciphering the cellular mechanisms that underlie the onset of infection and its ability to adapt to the food processing environment. Although information is available on the presence of *L. monocytogenes* in many environmental niches including soil, water, plants, foodstuff and animals, understanding the ecology of *L. monocytogenes* in outdoor environments has received less attention. Soil is an environmental niche of pivotal importance in the transmission of this bacterium to plants and animals. Soil composition, microbial communities and macrofauna are extrinsic edaphic factors that direct the fate of *L. monocytogenes* in the soil environment. Moreover, farming practices may further affect its incidence. The genome of *L. monocytogenes* presents an extensive repertoire of genes encoding transport proteins and regulators, a characteristic of the genome of ubiquitous bacteria. Postgenomic analyses bring new insights in the process of soil adaptation. In the present paper focussing on soil, we review these extrinsic and intrinsic factors that drive environmental adaptation of *L. monocytogenes*.

## Introduction

**Figure 1:**
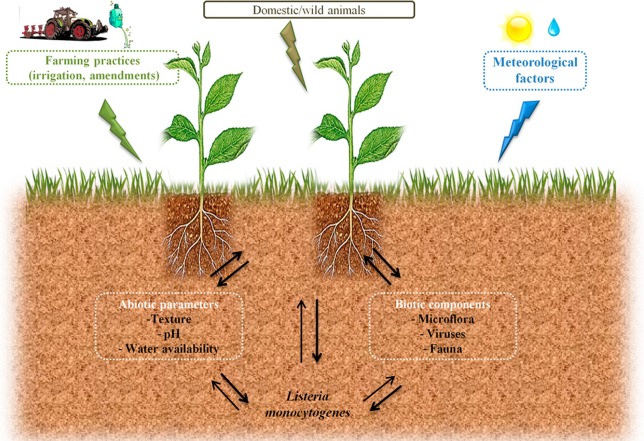
**Possible routes of transfer and circulation of *L. monocytogenes* in the farm environment and factors which can affect its survival in soil**.

Circulation of zoonotic and Human pathogens within the biosphere is a major health issue. Agroecosystems may participate to the transmission of pathogens to the food chain through production of contaminated raw products. However, as illustrated in Figure 1, understanding conditions that will trigger such contaminations or, on the opposite that will limit risks of contamination is difficult in the face of the complexity of the ecology of microorganisms. *Listeria monocytogenes* is the agent of listeriosis, a food-borne illness. Health effects range from flu-like symptoms with vomiting and diarrhea in healthy adults to life-threatening diseases such as meningitis and septicaemia in vulnerable people and spontaneous abortion in pregnant women. In the light of these health hazards, this bacterium received much attention in order to understand the physiopathology of listeriosis. As a matter of fact, *L. monocytogenes* has become a paradigm for the study of intracellular pathogens. Similarly, the mechanisms that underlie its ability to persist in foodstuff and in the food manufacturing environment have been documented. However, the ecology of *L. monocytogenes* in outdoor environments is only partially understood. The objective of this review is to present the state of the art regarding extrinsic and intrinsic factors that shape the ecology of *L. monocytogenes* in the soil environment.

Understanding causal factors of agroecosystems invasion by human pathogens is required in a period of adaptation of farming practices to global changes in order to avoid emergence of health hazards related to increased circulation of pathogens.

### Data available on the occurrence of *L. monocytogenes* in soil

Several reports on the incidence of *L. monocytogenes* in soil are available (Table 1). The pioneering work by Welshimer presented the first evidence that soil is an environmental niche for *L. monocytogenes* and occurrence of the bacterium was observed in a third of the 12 sampled farms (Welshimer, 1960; Welshimer and Donker-Voet, 1971). This was further supported in the seventies by the work of Weis and Seeliger. These authors surveyed the occurrence of *L. monocytogenes* in 746 soil samples collected in Southern Germany. A total of 160 strains were isolated and the overall incidence of *L. monocytogenes* was 21.4% (Weis and Seeliger, 1975). In this study, variations from 8.4 to 44.0 in the percentage of positive samples were observed according to the type of culture in the field and the highest incidence was recorded for uncultivated fields and meadows. Higher prevalence in meadows and uncultivated fields (30.8%) compared to cultivated fields (8.3%) was confirmed later (Dowe et al., 1997). Similarly, in a recent survey of the distribution of the *Listeria* species in urban and natural environments in the US, while overall prevalence in soil was 19% in natural environments and 30% in urban environments, spatial variation of the prevalence of *Listeria* sp. was observed within each category (Sauders et al., 2012). Temporal variation of the detection of *L. monocytogenes* has been documented after sampling at the same site (Weis and Seeliger, 1975; Sauders et al., 2012). Moreover, prevalence differed significantly according to the season and category of environment. Indeed, it was highest during summer in natural environments but lowest at this time of the year in urban environments (Sauders et al., 2012). In another report of a 3-year survey on fruit and vegetable farms, prevalence was higher in winter except during 1 year (Strawn et al., 2013) and correlation between season and occurrence is not a clear-cut. Incidence of *L. monocytogenes* in soil samples collected from small ruminant and cattle farms has also been recorded (Garcia et al., 1996; Nightingale et al., 2004; Fox et al., 2009).

**Table 1:** ***Listeria monocytogenes* occurrence in soil and culture-based procedure used for its isolation**.

**References**	**Enrichment procedure**	**Enrichment broth**	**Condition of incubation**	**Percentage of soil detected as positive**
Welshimer and Donker-Voet, 1971	2-step	BHI^*^	4°C, 3.5–5 months	92% of farm samples^**^
			37°C, 24 h	86% of the non-agricultural soil samples^**^
Weis and Seeliger, 1975	1-step	Lehnert + acriflavin (10 μg/ml)	22°C, 7 days	From 5.2–51.4% according to vegetation cover
MacGowan et al., 1994	2-step	LEB^***^	30°C, 48 h, and 7 days	0.7% soils from urban origin
Garcia et al., 1996	2-step	Two-step enrichment		8.3% ewe's farmyard
Dowe et al., 1997	2-step	LEB/Frazer broth	30°C, 48 h/35°C, 24–48 h	8.3% in carrot fields
				30.8% in adjacent meadows
Nightingale et al., 2004	1-step	LEB	30°C, 48 h	24% of samples from ruminant farms
Fox et al., 2009	1-step	LEB	30°C, 48 h	3% of soils from dairy farms
Sauders et al., 2012	1-step	LEB	30°C, 24–48 h	7.1% of soils collected from pristine field areas
				0.4% of soils collected from pristine forest areas
				10.7% of soils collected from urban environments
Locatelli et al., 2013a	2-step	LEB + modified LEB	37°C, 24–48 h	17% of field samples were positive in at least one of the three enrichment procedure
	2-step	Frazer broth	37°C, 48 h	
	1-step	Frazer broth	4°C, 2 months	
Strawn et al., 2013	1-step	LEB supplemented with a *Listeria* selective enrichment supplement	30°C, 24–48 h	9% of soils collected from fruit and vegetable farms

* Brain heart infusion,

** These figures should be considered cautiously as taxonomy of the genus Listeria has drastically evolved since the 70ths,

*** Listeria enrichment broth.

In these surveys, detection always required selective enrichment. When available, the actual concentration of *L. monocytogenes* reported from soil samples is low (MacGowan et al., 1994; Dowe et al., 1997). It suggests that the population of *L. monocytogenes* in soil is generally low. This was confirmed in a French nationwide survey where a PCR assay was performed on a collection of 1232 soil DNA for the specific detection of *L. monocytogenes* (Locatelli et al., 2013a). All samples were below the detection limit of 10^4^ g^−1^ of dry soil. Interestingly, a comparison of cultivation-based and molecular detection on a subset of 53 fresh soil samples showed that the incidence of culturable *L. monocytogenes* was 17% but only one of these samples was positive for the molecular detection of *L. monocytogenes* and its population was quantified at 2.88 10^4^ g^−1^ of dry soil (Locatelli et al., 2013a).

These surveys clearly demonstrate that soil is an environmental niche of *L. monocytogenes* but its population is generally low. Temporal and spatial variations of its occurrence point out that environmental factors drive the fate of *L. monocytogenes* in soil.

### Edaphic factors that affect its survival

Soil is an extremely complex and heterogeneous environment composed of minerals, organic matter, lacy plant roots and a complex biota including microorganisms, viruses, mesofauna and macrofauna. All of these are constantly interacting and soil may be regarded as an environment under dynamic equilibrium (Kogel-Knabner et al., 2008). Soil is characterized by complex food webs influenced by below-ground and above-ground processes (Wardle, 2006). As such, deciphering environmental drivers that impact the occurrence of *L. monocytogenes* in soil is extremely hard as these drivers are interconnected.

One of the conclusions that can be drawn from the results of the surveys presented above is that the nature of the soil affects the occurrence of *L. monocytogenes*. However, in these reports, indications of the soil characteristics are coarse. This makes it difficult to extract significant information regarding the relation between soil's characteristics and the presence of *L. monocytogenes*.

Investigations on listerial population dynamics after inoculation of soil microcosms (Table 2) evidenced that water availability is critical to its survival (Welshimer, 1960; Picardbonnaud et al., 1989; Vanrenterghem et al., 1991; Dowe et al., 1997). The type of soil affects population dynamics. Sandy soil represents an environment less favorable for *L. monocytogenes* than sandy loam and clay loam soils (Dowe et al., 1997); lower survival in clay soil than in fertile garden soil was also reported (Welshimer, 1960). A drawback of these studies is that only one soil of each type was used. A statistical approach to the analysis of *L. monocytogenes* survival in soils was recently performed to circumvent this limitation of the published data (Locatelli et al., 2013b). Data on the survival of *L. monocytogenes* in 100 soil microcosms was analyzed in the light of a comprehensive and detailed characterization of the soil samples (Locatelli et al., 2013b). These results confirmed that survival is dependent of the type of soil. Survival up to 84 days was observed in 71% of the soils tested while survival did not exceed 14 days in the rest of the soil microcosms. Variance partitioning explained this short-term survival by the soil chemical properties, especially the basic cation saturation ratio. The long-term survival could be related to the soil texture and especially clay content. The report by Locatelli et al. and others evidenced that the pH of the soil is a major driver of the fate of *L. monocytogenes* in soil (Weis and Seeliger, 1975; Sidorenko et al., 2006; McLaughlin et al., 2011; Locatelli et al., 2013b).

**Table 2:** ***L. monocytogenes* population dynamics after inoculation of soil microcosms**.

**References**	**Soil type**	**Condition of incubation**	**Inoculum (CFU/g)**	**Population dynamics at the end of the experiment**
**NON-STERILIZED SOILS**				
Welshimer, 1960	“Fertile”	30°C, 295 days	1.10^8^	Decreased but still detectable throughout the incubation
	“Clay”			Approached the zero level around Day 195
Vanrenterghem et al., 1991	Sandy loam	15°C, 8 weeks	1.10^5^	Intermittently detected during 6 weeks after incubation
Dowe et al., 1997	Clay loam, sandy loam and sandy	25–30°C, 32 days	1.10^2^	Increase to 10^4^ in clay loam and sandy loam—stable in sandy soil
			1.10^6^	Decrease to 10^4^ in clay loam and sandy loam—less than10^3^ in sandy soil
Sidorenko et al., 2006	Brown podzolic	20–22°C, 7 days	1.10^2^	Stable
	Forest			Undetectable after Day 2
	Urban			Increase to 10^4^
McLaughlin et al., 2011	Forest	8°C, 14 days	1.10^6^	1.2 10^3^ at the end of the experiment
		25 and 30°C, 14 days		Sharp decrease and undetected by Day 8
Locatelli et al., 2013b	100 contrasted soils	20°C, 84 days	1.10^6^	Declined with time
**STERILIZED SOILS**				
Dowe et al., 1997	Clay loam, sandy loam and sandy	25–30°C, 32 days	1.10^2^	Increase to 10^5^in clay loam—10^4^in sandy loam and sandy
			1.10^6^	Stable at 10^4^in clay loam and sandy loam—down to 10^5^in sandy soil
McLaughlin et al., 2011	Forest	25°C, 14 days	1.10^6^	Over 1 log increase within 2 days, then decrease down to 10^6^
Piveteau et al., 2011	Loamy	25°C, 1 year	1.10^5^	Over 3 log increase in 2 days then slow decline to 10^6^ cfu.g^−1^
Locatelli et al., 2013b	9 contrasted soil	20°C, 84 days	1.10^6^	a rise (1 to 3 log) in 3 soils—Decline in 6 soils

### Role of soil microflora, microfauna, mesofauna and macrofauna

Soil is a rich and complex biotic environment that generates massive interest because of the immense diversity it harbors and the many services associated (Nannipieri et al., 2003; Decaens, 2010; Griffiths and Philippot, 2013). The impact of indigenous microorganisms on the fate of *L. monocytogenes* in soil has been addressed experimentally in microcosms. In these experiments, population dynamics of *L. monocytogenes* were compared in autoclaved and non-autoclaved soil microcosms. The results clearly demonstrated that autoclaving facilitates implantation of *L. monocytogenes* in soil as growth was observed in autoclaved soil while the population decreased in non-autoclaved soil (Welshimer, 1960; Botzler et al., 1974; Vanrenterghem et al., 1991; Dowe et al., 1997; McLaughlin et al., 2011). However, this effect of the soil microflora cannot be observed in soils with low pH as a decline of the listerial population was observed even in sterilized soils (Locatelli et al., 2013b). Interestingly, this antagonistic effect depends on the diversity and the structure of the soil microflora (Vivant et al., 2013). In this report, experimental erosion of diversity clearly showed that loss of biotic diversity resulted in a better survival of *L. monocytogenes* in soil.

In the complex food web of the soil environment, interactions of *L. monocytogenes* with microfauna, mesofauna and macrofauna are most likely and this assumption is supported by lab experiments.

Interaction of *L. monocytogenes* with bacteriophagous protozoans has been documented (Ly and Muller, 1990; Barker and Brown, 1994; Harf, 1994; Gourabathini et al., 2008). Once internalized by endocytosis, it can grow within the cytoplasm of *Acanthamoeba* sp. and *Tetrahymena pyriformis* and eventually causes lysis and death of the host within 8–14 days (Barker and Brown, 1994; Harf, 1994). In a study focused on *Glaucoma* sp. isolated from lettuce, *Tetrahymena* sp. isolated from spinach and *Tetrahymena sp*. isolated from soil, intracellular survival of *L. monocytogenes* was confirmed (Gourabathini et al., 2008). Finally, *L. monocytogenes* survives endocytosis by *Acanthamoeba castellanii*; this protozoan could actually enhance growth of the bacterium while feeding on various species of *Listeria* was not lethal to the protozoan (Zhou et al., 2007). These are indirect indications that protozoans can act as vectors of dispersion of *L. monocytogenes* in soil. However, other reports suggest that the ability of *L. monocytogenes* to survive after ingestion by protozoans may be strain and/or species specific as killing of *L. monocytogenes* by *Acanthamoeba polyphaga*, *Acanthamoeba castellanii* and *Acanthamoeba lenticulata* has been reported (Akya et al., 2009, 2010).

Reports on the role of free living nematodes feeding on bacteria as vectors of dispersion of food-borne pathogens are available in the literature (Anderson et al., 2003, 2006; Caldwell et al., 2003; Gibbs et al., 2005; Thomsen et al., 2006; Forrester et al., 2007; Zhou et al., 2007; Guha et al., 2013). *Ceanorhabditis elegans* can feed on *L. monocytogenes*, moreover, in an agar plate assay, presence of live cells in the gut and excrement was confirmed and shedding of the pathogen was evidenced (Anderson et al., 2003, 2006; Caldwell et al., 2003; Guha et al., 2013). Interestingly, shedding by *Diploscapter* sp., a bacteriovorous soil nematode, was observed in soil amended with composted turkey manure (Gibbs et al., 2005). Feeding on *L. monocytogenes* may have deleterious effects on *C. elegans* (Forrester et al., 2007) and killing of the nematode was demonstrated experimentally (Thomsen et al., 2006) but others did not report killing (Guha et al., 2013) and this remains an open question.

Information on interactions between *L. monocytogenes* and soil mesofauna and macrofauna is scarce (Kuzina et al., 2001; Mansfield et al., 2003; Sezen et al., 2004; Lapanje et al., 2010). However, these reports support the idea that several species of the soil meso- and macrofauna could act as reservoirs and vectors. Indeed, *Listeria* sp. was detected in the gut of the isopod *Porcelio scaber* (Lapanje et al., 2010) and in the gut of the Diptera *Anastrepha ludens* (Kuzina et al., 2001). Infection of another Diptera, *Drosophila melanogaster*, by *L. monocytogenes* has been demonstrated experimentally (Mansfield et al., 2003). In this work, intra-host multiplication was observed resulting in killing of the infected fruit flies within 6–8 days. *Listeria* sp. has been isolated from the bacterial flora of the Coleoptera *Agelastica alni* with a frequency of 18% (Sezen et al., 2004). Finally, *Listeria* sp. was detected in half of 10 wormfarms sampled but *L. monocytogenes* was not isolated in any of them (Smith, 2001). Considering the diversity of the soil fauna (Decaens, 2010), one can assume that many species probably interact with *L. monocytogenes*. Elucidation of these complex interactions in the soil food web would bring a new understanding of the ecology of this food-borne pathogen. Moreover, wild animals including mammals and birds must be considered as potential zoonotic reservoirs of the pathogen (Weis and Seeliger, 1975; Yoshida et al., 2000; Lyautey et al., 2007b) and may participate to its transfer to soil.

### Farming practices and transfer into the soil (compost, sewage sludges, reclaimed waste)

Telluric occurrence of *L. monocytogenes* and other food-borne pathogens raises health issues in cultivated fields and grazing pastures as soil may be a vector of pathogens to cultivated plants and farmed animals. Hence, cattle and small ruminants are reservoirs of *L. monocytogenes* (Garcia et al., 1996; Nightingale et al., 2004; Fox et al., 2009). There is a body of evidence that suggests that farming practices can directly impact on the circulation and implantation of *L. monocytogenes*.

Ensilage of contaminated crops may result in the increase of *L. monocytogenes* populations in the final feed that may become the vector of cattle listeriosis (Fenlon, 1985, 1986; Wiedmann et al., 1996).

Agricultural recycling of organic wastes without sanitation procedures is a route of transmission of *L. monocytogenes* to soil. Indeed, stored sewage sludges frequently contain *L. monocytogenes* at low levels (1–240 bacteria MPN g^−1^ dry matter) and their agricultural use as fertilizer without sanitation procedure may facilitate its transfer and implantation into soil (Watkins and Sleath, 1981; Alghazali and Alazawi, 1990; De Luca et al., 1998; Garrec et al., 2003). Per hectare, spreading of one to two tons of sludge would bring around 10^6^–10^8^ *L. monocytogenes* per year (Garrec et al., 2003).

The presence of *L. monocytogenes* in the faeces of farm animals has been recognized (Fenlon et al., 1996; Nicholson et al., 2004; Nightingale et al., 2004). In a case-control study involving a total of 52 bovine, goat and sheep farms, the frequency of detection varied from 22 to 33% in bovine farms and from 3 to 18% in goat and sheep farms (Nightingale et al., 2004). Survival during storage of faecal wastes is limited to a few weeks but daily inputs in the storage facilities of the farmyard could maintain a constant load of viable *L. monocytogenes* (Vanrenterghem et al., 1991; Hutchison et al., 2005). Land spreading of these untreated wastes can contribute to the transmission of *L. monocytogenes* to soil. In an experiment of spreading of artificially contaminated faecal wastes on a grass pasture, 64–128 days were required until the listerial population declined to undetectable levels (Hutchison et al., 2005). In a separate experiment maximum survival period varied from 4 days to over 32 days after land application according to the type of waste (Nicholson et al., 2005). Delaying incorporation of faecal waste after land application on agricultural soils could result in a decline in the population of the pathogen (Hutchison et al., 2004). Under laboratory conditions, survival in bovine manure-amended soil varied from 21 to 43 days and depended on the dose and on the temperature of incubation (Jiang et al., 2004).

Survival up to 56 days was observed in a climatic chamber experiment designed to investigate the fate of several pathogens after spreading of anaerobic digestion residues on grass crops (Johansson et al., 2005).

Statistical integration of survey data gives interesting insights into the environmental parameters affecting the probability of occurrence of the pathogen. Ivanek et al. found a strong association between weather, soil properties and the probability to detect *Listeria* sp. in soil (Ivanek et al., 2009). In a recent 2 year long longitudinal study of the occurrence of food-borne pathogens in 5 fruit and vegetable farms, landscape and meteorological factors were associated to the frequency of positive samples (Strawn et al., 2013). Overall prevalence of *L. monocytogenes* was 15% while in soil, the frequency of detection was 9%. This survey was exploited to develop a classification tree model. This evidenced that temperature, proximity to surface water, roads/urban development and pasture/hay grass, but also soil-related parameters (available water storage, soil organic matter) were environmental and topographic factors of importance to explain detection of *L. monocytogenes* (Strawn et al., 2013). Interestingly, in this survey, the frequency of detection of *L. monocytogenes* was highest in water samples. The quality of water used for land irrigation is of concern and transfer of pathogens through poor quality irrigation water is likely (Steele and Odumeru, 2004; Selma et al., 2007; Ijabadeniyi et al., 2011; Oliveira et al., 2011). Proximity to dairy farms is an environmental factor that influences detection of *L. monocytogenes* in watersheds (Sauders et al., 2006; Lyautey et al., 2007a). Wastewater treated effluents frequently carry *L. monocytogenes* (Watkins and Sleath, 1981; Alghazali and Alazawi, 1990; Steele and Odumeru, 2004; Paillard et al., 2005; Odjadjare et al., 2010; Moreno et al., 2011) with loads varying from 3 to 15 CFU.ml^−1^ (Alghazali and Alazawi, 1990) to over 10^3^ CFU.ml^−1^ (Odjadjare et al., 2010).

### Intrinsic factors in relation to the gene content/transcriptional regulation

Sequencing of the genome of *L. monocytogenes* yields insights into traits that permit persistence of the pathogen into soil. The description of the genome of *L. monocytogenes* EGD-e, the first sequenced strain, highlighted features relevant to the ubiquity of this species (Glaser et al., 2001). Indeed, genes encoding transport proteins represent a high proportion of the genome (11.6% of the genome of *L. monocytogenes* EGD-e) 26% of them are related to the transport of carbohydrates by phosphoenolpyruvate-dependent phosphotransferase systems. It is thus equipped to have access to multiple carbon sources from varying environments. The proportion of regulatory genes is also high (7.3%). Such an extensive repertoire of regulatory genes is expected from a versatile bacterium that thrives in many habitats. The role of some of them has been studied extensively, especially PrfA and VirR because of their importance during infection of the mammal host.

At the moment, over 20 genomes of representatives of the genus *Listeria* are available (4 non *L. monocytogenes* and 24 *L. monocytogenes*) (http://img.jgi.doe.gov/cgi-bin/w/main.cgi?). These are circular chromosomes ranging from 2.7 to 3.0 Mb in length. A characteristic of these genomes is their high degree of synteny (den Bakker et al., 2012, 2013). These comparative genomic analyses identified around 4400 genes in the pan-genome of *L. monocytognes*. Among the putative protein coding genes (between 2330 and 2465 genes) about 80% are within the core genome. This rather high proportion of core genes relative to several other species is consistent with a stable species backbone and a limited accessory genome (from 323 to 753 genes per strain) (den Bakker et al., 2013). Interestingly, genes involved in transport and metabolic processes are overrepresented in both core and accessory genomes (den Bakker et al., 2012). *In silico* comparison of the representation of Clusters of Orthologous Groups within the genomes of the genus *Listeria*, *Bacillus*, *Lactobacillus*, *Staphylococcus* and *Streptococcus* (Markowitz et al., 2012) illustrates the richness of all listerial genomes, similar to *Bacillus* genomes, in genes coding transport proteins and transcriptional regulators (Table 3). This could facilitate adaptation to various habitats, a trademark of the bacteria of the genus *Bacillus* and *Listeria*. Within the genus *Listeria*, the proportion of genes coding transport proteins, histidine kinases and transcriptional regulators are similar (Table 4). Noteworthy is the overrepresentation of PTS systems in both core and accessory genomes (Glaser et al., 2001; Doumith et al., 2004; Deng et al., 2010; den Bakker et al., 2013). Such a pool of carbohydrate transporters and the ability to metabolize an extended range of these carbon sources could represent a fitness advantage under the control of selective pressure in the environment and *in vivo* (den Bakker et al., 2013). Under biotic conditions, the diversity of PTS systems and their possible functional redundancy could attenuate the efficacy of PTS-triggering bacteriocins (Diep et al., 2007; den Bakker et al., 2013) produced by neighboring bacteria (den Bakker et al., 2013).

**Table 3:** **Median number of genes from functional categories transport, histidine kinase and transcriptional regulator across selected genomes**.

**Function**	**Distribution in taxonomic unit**					
	**Firmicutes**	***Listeria***	***Bacillus***	***Lactobacillus***	***Staphylococcus***	***Streptococcus***
	***N*** = **521**	***N*** = **28**	***N*** = **70**	***N*** = **48**	***N*** = **50**	***N*** = **90**
Transport	257 (204)	320 (311)	466 (466)	208 (208)	266 (266)	216 (247)
Carbohydrate transport	20 (7)	23 (23)	32 (27)	13 (21)	7 (7)	17 (10)
Amino acid transport	43 (44)	40 (39)	80 (63)	48 (37)	44 (44)	41 (36)
PTS	27 (24)	86 (86)	29 (29)	28 (5)	25 (25)	41 (41)
Histidine kinase	15 (9)	15 (15)	50 (35)	8 (6)	15 (15)	9 (9)
Transcriptional regulator	89 (65)	142 (144)	204 (170)	70 (70)	77 (55)	68 (61)

N is the number of genomes considered for each taxonomic unit. The mode is presented in brackets.

**Table 4:** **Median number of genes from functional categories transport, histidine kinase and transcriptional regulator across the genus *Listeria* compared to *Bacillus subtilis* and Firmicutes**.

**Function**	**Firmicutes**	***Bacillus subtilis***	*Listeria* sp^*^	***L. monocytogenes***		
	*****N*** = **521****	*****N*** = **12****	*****N*** = **4****	**Lineage I**	**Lineage II**	**Lineage III**
				***N*** = **10**	*****N*** = **10****	*****N*** = **4****
Transport	257	413 (371–650)^**^	315 (281–327)	320 (299–335)	330 (300–333)	311 (298–311)
Carbohydrate transport	20	44 (36–61)	23 (19–24)	23 (22–25)	23 (23–25)	22 (22–23)
Amino acid transport	44	62 (53–95)	38 (34–45)	39 (39–44)	40 (39–45)	39 (38–42)
PTS	27	29 (27–34)	85 (59–90)	83 (81–92)	87 (86–108)	80 (77–94)
Histidine kinase	15	37 (34–77)	15 (14–15)	15 (14–16)	16 (15–16)	15 (14–15)
Transcriptional regulator	89	181 (169–206)	132 (123–140)	142 (137–144)	146 (142–151)	139 (136–140)

N is the number of genomes considered for each taxonomic unit.

* L. welshimeri, L. innocua, L seeligeri, L. ivanovii.

** In brackets are the minimum and maximum numbers of genes found in the genome.

Extrachromosomal DNA has been detected in many *L. monocytogenes* strains (Lebrun et al., 1992, 1994; McLauchlin et al., 1997; Lemaitre et al., 1998; Katharios-Lanwermeyer et al., 2012). Overrepresentation of plasmids in strains from food and saprophytic environments in comparison to those from clinical cases was reported (Lebrun et al., 1992; McLauchlin et al., 1997; Kuenne et al., 2010). A number of diverse mobile genetic elements and genes involved in heavy metal resistance (cadmium, copper, arsenite) as well as multidrug efflux and oxidative stress response are generally found on plasmids isolated from representatives of the genus *Listeria* (Kuenne et al., 2010). The presence of these plasmids could assist survival in food processing environments. Recently, a unique type of plasmid was found in some strains of lineage IV responsible of caprine listeriosis outbreaks (den Bakker et al., 2012). Unlike what was discussed above, this plasmid does not carry genes involved in resistance to antibiotic or heavy metals but internalin-like genes that might facilitate invasion of the caprine host. Still, the role of large plasmids in the ecology of *L. monocytogenes* is poorly understood although it is probably relevant to explain the fitness of isolates.

Information on the molecular mechanisms that underlie growth and persistence of *L. monocytogenes* in soil is scarce. Transcriptome analysis during adaptation to the soil environment showed that *L. monocytogenes* mobilized its repertoire of genes coding transporters (phosphoenolpyruvate-dependent phosphotransferase systems and ABC transporters) and enzymes involved in catabolism of specific carbohydrates (β-glucosidases; chitinases) (Piveteau et al., 2011). It is consistent with the requirement to utilize the carbon, nitrogen and energy sources available in the soil in order to persist in this environment. Overrepresentation of genes from the SigmaB regulon was noticed (Piveteau et al., 2011). The role of SigmaB for the survival and adaptation of *L. monocytogenes* in soil has been demonstrated experimentally (Gorski et al., 2011). Indeed, SigmaB regulates the response and tolerance to most stresses in *L. monocytogenes*, including those that can be encountered in soil. A complex regulatory network underlies the ability of *L. monocytogenes* to thrive in diverse habitats and to respond efficiently to ever changing environmental conditions including transition from saprophytic to intracellular lifestyles (Chaturongakul et al., 2008). As discussed above, *L. monocytogenes* has the ability to avoid predation by bacteriophagous protozoans and bacteriovorous nematodes. Expression of virulence factors could be a fitness advantage during interaction with soil protozoans and other soil dwelling organisms. For example, defective mutants of *actA* and *prfA* are impaired during infection of *Drosophila melanogaster* (Mansfield et al., 2003). Moreover, in this study, expression of *actA* was evidenced at 25°C in insect cells. Similarly, listeriolysin O (LLO) is involved in the interaction with *Tetrahymena pyriformis* and *hly* deficient mutants fail to cause mortality of the ciliate (Pushkareva and Ermolaeva, 2010). As such, expression of some of the identified virulence factors could contribute to the overall fitness of *L. monocytogenes* in the soil ecosystem.

### Biodiversity and incidence in soil

One of the trademarks of the species *L. monocytogenes* is the wide range of environments where it is found. However, the ecological significance of the systematic definition of bacterial species is not straightforward. The concept of ecotype aims at synthesizing systematic, ecology and evolution in a new paradigm for the understanding of the ecology of microorganisms (Cohan, 2006; Cohan and Koeppel, 2008). Ecotypes are defined as “the smallest groups that (i) show a history of coexistence as separate, ecologically distinct lineages, as inferred from community ecology (or an equivalent sequence-based approach) and (ii) show a prognosis for future coexistence, as inferred from the ecological distinctness of the groups in nature” (Cohan, 2006). Whether or not ecotypes exist in the species *L. monocytogenes* is an open question. The phylogenetic structure of *L. monocytogenes* is complex. Isolates are grouped in four lineages and major clonal complexes are recognized (Nadon et al., 2001; Ragon et al., 2008). These major clonal complexes are distributed worldwide (Chenal-Francisque et al., 2011). Interestingly, there is evidence that distribution of clones and serotypes differ among clinical, food and environmental isolates (Wiedmann et al., 1997; Gray et al., 2004; Ward et al., 2004; Chenal-Francisque et al., 2011). However, a limitation of these studies is that the collections of isolates that were analyzed are not fully representative of the complex ecology of members of the species *L. monocytogenes*. Ribotyping of farm animal isolates of *L. monocytogenes* identified several subtypes (Gudmundsdottir et al., 2004; Nightingale et al., 2004). In a case study, Nightingale et al. showed that the incidence of one of the ribotypes was higher in soil while others were preferentially associated with faecal samples and animals listeriosis (Nightingale et al., 2004). In two recent publications, the authors compared the diversity of *Listeria* strains isolated from soil, water and vegetation from natural areas of the state of New York (USA) and from urban soil, water, vegetation samples and several surfaces found in cities (Sauders et al., 2006, 2012). Evidence also pointed out to higher detection of specific subtypes in specific sample sites. These results are in favor of the existence of ecotypes. However, others report on the widespread distribution of PFGE types regardless of their origin (Fugett et al., 2007).

As proposed by Cohan (Cohan, 2006), defining such ecotypes will require a clear demonstration that the ecology of isolates of the various sequence clusters is actually distinct.

## Conclusion

Soil is a complex ecosystem central to the function of the biosphere. Agricultural use of land may raise health issues. Indeed, soil may play a pivotal role in the transfer of Human pathogens to cultivated plants and farm animals and the subsequent contamination of foodstuff. As discussed in this review, *Listeria monocytogenes* is a human pathogen naturally present in soil. Indeed, intrinsic factors such as an extended repertoire of transport systems, especially PTS and transcriptional regulators underlie the ability of the members of this species to persist in the soil ecosystem. However, extrinsic factors affect their ability to survive in soil. These include edaphic parameters and the biotic environment which direct the fate of *L. monocytogenes*. The natural history of members of the species *L. monocytogenes* is complex, circulation of the pathogen between environments is still rather poorly understood and whether or not ecotypes exist remains an open question.

Considering their ability to persist in soil, it is rather difficult to predict how anthropogenic-driven changes could modify their circulation and incidence in the biosphere. In a period of rapid global changes, when agriculture faces major adaptation challenges, understanding the ecology of Human pathogens in agroecosystems is necessary to forecast how their circulation and incidence may be affected. It is indeed a prerequisite to reassess health hazards. As a matter of fact, *L. monocytogenes* should be considered as a useful model organism for this purpose.

### Conflict of interest statement

The authors declare that the research was conducted in the absence of any commercial or financial relationships that could be construed as a potential conflict of interest.
